# Supplementary material for: Foliar nutrient concentrations of six northern hardwood species responded to nitrogen and phosphorus fertilization but did not predict tree growth
Source: PeerJ. 2022 Apr 21;10:e13193. doi: 10.7717/peerj.13193 (PMC9035280; doi:10.7717/peerj.13193)
Supplement: Supplemental Information 1 [file peerj-10-13193-s001.docx]

**Appendix A. Timing of foliage collection by stand and species. Pre-treatment and post-treatment sampling dates are separated by an ampersand.**

| **Stand** | **BE** | **PC** | **RM** | **SM** | **WB** | **YB** |
| --- | --- | --- | --- | --- | --- | --- |
| **C1** | ’09/’10 & ’15/’16 | ’09/’10 & ’16 |  |  | ’09/’10 & ’16 |  |
| **C2** | ’09/’10 & ’14 | ’09/’10 & ’14 | ’09 & ’14 |  | ’09/’10 & ’14 | ’10 & ’14 |
| **C4** | ’08/’09/’10 & ’15 | ’08/’09/’10 & ’16 | ’08/’09 & ’15 |  | ’08/’09/’10 & ’16 | ’08/’09/’10 & ’16 |
| **C6** | ’08/’09/’10 & ’15 | ’09/’10 & ’16 | ’08/’09 & ’15 |  | ’08/’09/’10 & ’16 | ’08/’09/’10 & ’16 |
| **C8** | ’08/’09/’10 & ’15 |  |  | ’08/’09/’10 & ’15 |  | ’08/’09/’10 & ’16 |
| **C9** | ’08/’09/’10 & ’15 |  |  | ’08/’09/’10 & ’15 |  | ’08/’09/’10 & ’16 |
| **HBM** | ’10 & ’15 |  | ’08/’09 & ’15 |  | ’08/’09/’10 & ’16 | ’08/’09/’10 & ’16 |
| **HBO** | ’08/’09/’10 & ’15 |  |  | ’08/’09/’10 & ’15 |  | ’08/’09/’10 & ’16 |
| **JBM** | ’10 & ’15 | ’08/’09/’10 & ’16 |  | ’08/’09/’10 & ’15 | ’08/’09/’10 & ’16 | ’08/’09/’10 & ’16 |
| **JBO** | ’08/’09/’10 & ’15 |  |  | ’08/’09/’10 & ’15 |  | ’08/’09/’10 & ’16 |
