# Supplementary material for: Foliar nutrient concentrations of six northern hardwood species responded to nitrogen and phosphorus fertilization but did not predict tree growth
Source: PeerJ. 2022 Apr 21;10:e13193. doi: 10.7717/peerj.13193 (PMC9035280; doi:10.7717/peerj.13193)
Supplement: Supplemental Information 2 [file peerj-10-13193-s002.docx]

**Appendix B. Pre-treatment foliar N concentrations, foliar P concentrations, foliar N:P, foliar Ca concentrations, foliar Mg concentrations, and foliar K concentrations by species (mean ± SE across stands).**

| **Treatment** | **N (mg/g)** | **P (mg/g)** | **N:P** | **Ca (mg/g)** | **Mg (mg/g)** | **K (mg/g)** |
| --- | --- | --- | --- | --- | --- | --- |
| American beech | 23.8 ± 0.8 | 1.09 ± 0.03 | 21.8 ± 0.5 | 6.58 ± 0.47 | 1.39 ± 0.08 | 7.70 ± 0.46 |
| Pin cherry | 27.1 ± 0.8 | 1.44 ± 0.03 | 18.4 ± 0.6 | 10.18 ± 0.55 | 2.52 ± 0.09 | 11.87 ± 0.52 |
| Red maple | 18.9 ± 0.9 | 1.16 ± 0.04 | 15.8 ± 0.6 | 5.67 ± 0.60 | 1.23 ± 0.10 | 6.25 ± 0.56 |
| Sugar maple | 19.3 ± 0.8 | 1.06 ± 0.03 | 18.2 ± 0.6 | 7.15 ± 0.56 | 1.15 ± 0.10 | 7.55 ± 0.52 |
| White birch | 23.5 ± 0.8 | 1.11 ± 0.03 | 21.1 ± 0.6 | 6.42 ± 0.53 | 1.58 ± 0.09 | 8.03 ± 0.50 |
| Yellow birch | 24.9 ± 0.8 | 1.26 ± 0.03 | 19.6 ± 0.5 | 8.96 ± 0.48 | 2.15 ± 0.08 | 8.92 ± 0.47 |
